# Mild-moderate alcohol consumption and diabetes are associated with liver fibrosis in patients with biopsy-proven MASLD

**DOI:** 10.3389/fphar.2024.1437479

**Published:** 2024-07-31

**Authors:** Ang Huang, Cailun Zou, Zhe Dai, Ying Sun, Jing Wang, Shuhong Liu, Lin Han, Songhai Chen, Qingsheng Liang, Chunyan Wang, Yingjie Zhuang, Tong Dang, Binxia Chang, Yijin Wang, Zhengsheng Zou

**Affiliations:** ^1^ Department of Hepatology, The Fifth Medical Center of PLA General Hospital, Beijing, China; ^2^ Department of Gastroenterology and Hepatology, The First Medical Center of PLA General Hospital, Beijing, China; ^3^ Department of Gastroenterology, China-Japan Friendship Hospital, Beijing, China; ^4^ School of Medicine, Southern University of Science and Technology, Shenzhen, Guangdong, China; ^5^ Inner Mongolia Institute of Digestive Diseases, The Second Affiliated Hospital of Baotou Medical College, Inner Mongolia University of Science and Technology, Baotou, China; ^6^ Department of Pathology and Hepatology, The Fifth Medical Center of PLA General Hospital, Beijing, China; ^7^ Department for Disease Control and Prevention, The Fifth Medical Center of Chinese PLA General Hospital, Beijing, China

**Keywords:** MASLD, metabolic dysfunction-associated steatotic liver disease, mild-moderate alcohol consumption, fibrosis stage, type 2 diabetes mellitus, multivariate logistic regression

## Abstract

**Background:**

It is unclear whether patients with metabolic dysfunction-associated steatotic liver disease (MASLD) are allowed variable low levels of alcohol. This study aimed to evaluate the effect of mild-moderate alcohol consumption on the biochemical and histological characteristics of patients with MASLD.

**Methods:**

Alcohol consumption was assessed in 713 patients with steatotic liver disease (SLD) who underwent liver biopsy. Non-drinking, mild-moderate drinking, and excessive drinking were defined as 0 g/day, 1-<20 g/day, and >20 g/day for women and 0 g/day, 1-<30 g/day, and >30 g/day for men, respectively. Liver biopsies were scored according to the NASH CRN system.

**Results:**

A total of 713 participants (median age 39.0 years and 77.1% male) with biopsy-proven SLD were enrolled, including 239 nondrinkers, 269 mild-moderate drinkers and 205 excessive drinkers. Excessive drinking was associated with increased risks for lobular inflammation and liver fibrosis compared to nondrinkers and mild-moderate drinkers. Compared with non-drinkers, mild-moderate drinkers had significantly lower odds for steatosis (OR = 0.60, 95% CI = 0.38–0.93, *p* = 0.025), hepatocellular ballooning (OR = 0.52, 95% CI = 0.29–0.91, *p* = 0.020) and fibrosis (OR = 0.50, 95% CI = 0.31–0.81, *p* = 0.005). However, in non-excessive drinkers with type 2 diabetes mellitus (T2DM), there was no association between mild-moderate alcohol consumption and liver fibrosis (OR = 0.562, 95% CI = 0.207–1.530, *p* = 0.257).

**Conclusions:**

Mild-moderate alcohol consumption might be protective against liver fibrosis in MASLD patients, which is modified by the presence of T2DM. However, further longitudinal studies are needed to determine the effect of ongoing alcohol consumption on disease severity.

## Introduction

Metabolic dysfunction-associated steatotic liver disease (MASLD), formerly named nonalcoholic fatty liver disease (NAFLD), is already a leading cause of liver dysfunction and chronic liver disease worldwide and is associated with a burdening unmet clinical need (Younossi and Henry, 2016; Younossi et al., 2018; Xiao et al., 2019; Eslam et al., 2020; Chen et al., 2021). Liver fat accumulation is the hallmark of MASLD, which includes a spectrum of conditions ranging from simple steatosis to liver inflammation, liver fibrosis, and even liver cancer. Over the past decade, the incidence of MASLD has increased worldwide, surpassing viral infection as the chief etiology (Paik et al., 2020a; Zhou et al., 2020). Liver fibrosis related to MASLD has increasingly become an important cause of cirrhosis, liver failure, and hepatocellular carcinoma (Paik et al., 2020b; Collaborators, 2020; Golabi et al., 2021; Yip et al., 2022), leading to a major threat to public health. Currently, liver biopsy is the gold standard for diagnosing and staging MASLD.

Alcohol and obesity are the main risk factors for fibrosis in patients with MASLD, and they frequently coexist (Aberg et al., 2018). The influence of alcohol consumption on MASLD outcomes seems to be determined by the dose of alcohol; however, published data remain controversial (Di Castelnuovo et al., 2002; Ronksley et al., 2011). Some studies have reported that mild or moderate drinking has protective effects on patients with NAFLD/MASLD (Dunn et al., 2008; Dunn et al., 2012; Mitchell et al., 2018), while other studies have reported no association or even harmful effects (Kwon et al., 2014; Chang et al., 2019). With the increasing incidence of MASLD worldwide and the considerable number of alcohol drinkers among them, we aimed to investigate the association between different alcohol consumption doses and liver injury in a sample of well-characterized study participants with biopsy-proven MASLD.

## Patients and methods

### Patients

All subjects (aged 18 years or older) with liver biopsy-proven steatotic liver disease (SLD) were analyzed from January 2008 to December 2022 at the Fifth Medical Center of the Chinese PLA General Hospital and the Second Affiliated Hospital of Baotou Medical College. The clinical characteristics of all eligible patients were retrospectively obtained by retrieving electronic medical records during the study period. This is an observational, retrospective, cross-sectional study.

Patients with steatotic liver disease (SLD) were categorised into metabolic dysfunction-associated steatotic liver disease (MASLD), metabolic and alcohol related/associated liver disease (MetALD) and alcohol-associated liver disease (ALD) based on their level of alcohol consumption (Rinella et al., 2023). MASLD was diagnosed when hepatic steatosis (abdominal ultrasonography, computed tomography or magnetic resonance imaging) was present together with one of the following criteria (Rinella et al., 2023): 1) overweight or obesity (BMI ≥23 kg/m^2^ in Asians) or waist circumference ˃94/80 cm in Asian men and women, 2) Fasting serum glucose ≥5.6 mmol/L or HbA1c ≥ 5.7% or type 2 diabetes mellitus (T2DM) or treatment for T2DM, 3) blood pressure ≥130/85 mmHg or specific antihypertensive drug treatment, 4) plasma triglycerides ≥1.70 mmol/L or lipid lowering treatment, 5) plasma HDL-cholesterol ≤1.0 mmol/L for men and ≤1.3 mmol/L for women or lipid lowering treatment. MetALD was selected to describe those with metabolic dysfunction–associated steatotic liver disease, who consume average daily 20–50 g female, 30–60 g male, respectively.

**FIGURE 1:**
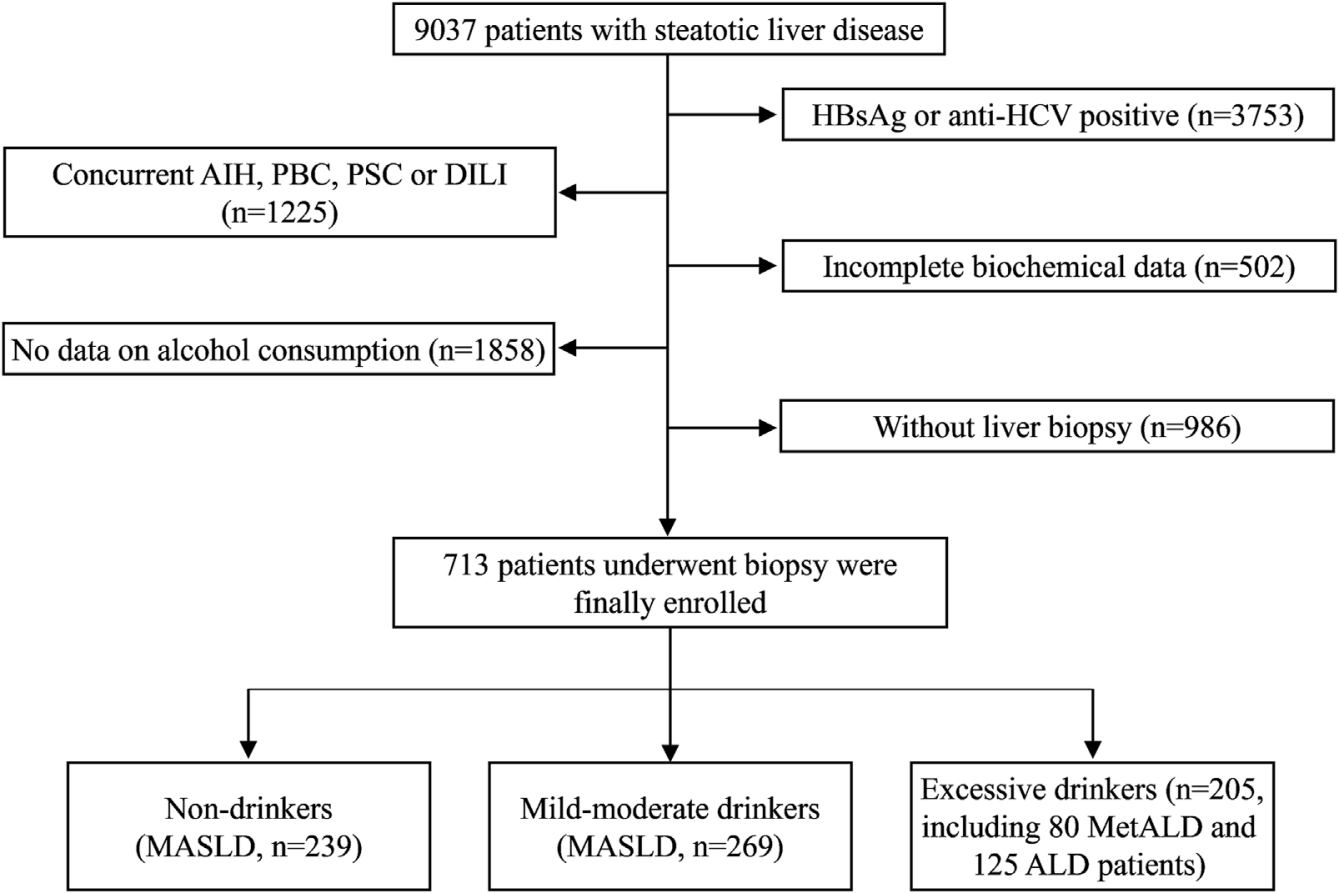
Flow chart of patients with MASLD enrolled in the study.

All of the enrolled patients met the following inclusion criteria: a) aged 18 years or older; b) had biopsy-proven fatty liver disease (steatosis ≥5%); c) did not receive specific pharmacologic therapy for MASLD/NAFLD within 6 months before enrollment; and the exclusion criteria were as follows: a) HBsAg positive or anti-HCV positive; b) incomplete biochemical data; c) no information on alcohol consumption; d) no liver biopsy; e) concurrent autoimmune diseases, such as autoimmune hepatitis (AIH), primary biliary cholangitis (PBC), or primary sclerosing cholangitis (PSC); and f) concurrent systemic illness and concurrent medications such as high-dose estrogens, corticosteroids, and amiodarone in the last 6 months. The procedure used for specimen collection is shown in Figure 1.

Criteria for the diagnosis of diabetes (Association, 2002): first, symptoms of diabetes and a casual plasma glucose ≥200 mg/dL (11.1 mmol/L). Casual was defined as any time of day without regard to the time since the last meal. The classic symptoms of diabetes include polyuria, polydipsia and unexplained weight loss. Second, fasting plasma glucose ≥126 mg/dL (7.0 mmol/L). Fasting was defined as no caloric intake for at least 8 h. Third, 2-h plasma glucose ≥200 mg/dL (11.1 mmol/L) during an oral glucose tolerance test. The test should be performed as described by the WHO, using a glucose load containing the equivalent of 75 g anhydrous glucose dissolved in water. Type 2 diabetes mellitus was diagnosed by experienced doctors during hospitalization and recorded in medical records.

### Study design

Demographic, routine biochemistry and histological data at inclusion were recorded. The clinical characteristics of the participants were first summarized and compared between different alcohol consumption doses based on three categories. We then analyzed the proportion of patients who developed severe histological outcomes and the frequency of each histological stage in the three groups. According to the association between MASLD and lipid and glucose metabolism disorders, we further investigated the burden of serum lipids and glucose profiles in subjects in different drinking categories. Univariate and multivariate logistic regression analyses were performed to identify the independent risk factors associated with the severity of histological liver injury in patients with MASLD.

Alcohol consumption was evaluated at study entry using the Alcohol Use Disorders Inventory Test (AUDIT) (Saunders et al., 1993). In this study, average alcohol consumption per day was calculated using the frequency and amount of alcohol consumed per drinking day. Non-drinking, mild-moderate drinking, and excessive drinking were defined as 0 g/day, 1-<20 g/day, and >20 g/day for women and 0 g/day, 1-<30 g/day, and >30 g/day for men, respectively (Chang et al., 2019). Non-drinkers who answered yes were considered previous drinkers currently not drinking alcohol and were excluded to limit the effect of former drinking.

This study was performed according to the ethical guidelines of the 1975 Declaration of Helsinki, as revised in 1983. This study was approved by the Ethics Committees of the Fifth Medical Center of Chinese PLA General Hospital (No. 2015-138-D). Written informed consent for liver biopsy was obtained from each relevant patient. Written informed consent for data collection was waived due to the study design.

### Histologic evaluation

Biopsy specimens were evaluated centrally by the Fifth Medical Center of Chinese PLA General Hospital for the following histologic features, according to the validated histologic scoring system by Kleiner et al. (2005). Liver biopsies were formalin-fixed, processed in paraffin, and stained with hematoxylin-eosin, Gordon-Sweet reticulin, Masson trichrome, erythrosine copper and Perls iron stains. Briefly, steatosis was graded on a 3-point scale: grade 1 (mild) = steatosis involving 5%–33% of hepatocytes, grade 2 (moderate) = 34%–66% steatosis, and grade 3 (severe) = >66% steatosis. A steatosis score ≥2 was considered to indicate significant steatosis. Lobular inflammation was graded on a 4-point scale: grade 0 = no foci, grade 1 ≤ 2 foci per 200 × field, grade 2 = 2–4 foci per 200 × field, and grade 3 = >4 foci per 200 × field. Lobular inflammation scores ≥2 were considered significant lobular inflammation. Hepatocellular ballooning was graded on a 3-point scale: grade 0 = none, grade 1 = few, and grade 2 = many. Ballooning scores >1 were considered to indicate significant ballooning. Fibrosis was assessed on a 5-point scale: stage 0 = none, stage 1 = perisinusoidal or periportal, stage 2 = perisinusoidal and portal/periportal, stage 3 = bridging fibrosis, and stage 4 = cirrhosis. A fibrosis score ≥2 was considered to indicate significant fibrosis.

### The relationship between alcohol consumption and serum lipid and glucose metabolism

Patients with MASLD were stratified according to the severity of hepatic steatosis (mild, moderate, or severe) and fibrosis (fibrosis stage 0–1, mild; fibrosis stage 2, moderate; and fibrosis stage 3–4, severe). Lipid profiles and glucose levels in the three groups with different drinking habits were assessed at the same hepatic steatosis and fibrosis status.

### Univariate and multivariate logistic regression analyses of alcohol use on steatosis, lobular inflammation, ballooning and fibrosis

Based on our clinical experience and reference to previous studies (Dunn et al., 2012; Ajmera et al., 2018; Mitchell et al., 2018; Chang et al., 2019), we selected the variables for the regression analyses. Univariate logistic regression analysis determined the independent predictors of histological outcomes (significant steatosis, lobular inflammation, ballooning and fibrosis) in MASLD patients. After conducting univariate analyses, variables with a *p*-value of < 0.1 were selected for multivariate logistic regression analysis.

### Statistical analysis

If the continuous variables were normally distributed, the data are presented as the mean ± standard deviation (mean ± SD). When they were not normally distributed, the median (interquartile range, IQR) was presented. Continuous variables were compared using Student’s t-test or analysis of variance if normally distributed and Mann‒Whitney U test or Kruskal‒Wallis H nonparametric test if not normally distributed. Categorical variables are expressed as frequencies with percentages. The categorical variables were analyzed by the R × C chi-square test. The risk factors associated with steatosis, fibrosis and lobular inflammation were assessed by univariate and multivariable logistic regression using the R package “glmnet” (Friedman et al., 2010). All statistical tests were conducted using R software, version 4.0.2 (R Foundation for Statistical Computing, Vienna, Austria; http://www.r-project.org/). Differences were considered significant when *p* < 0.05. All values are presented as the means with the corresponding standard errors.

## Results

### Study population

As shown in Figure 1, a total of 9,037 patients diagnosed with steatotic liver disease (hepatic steatosis identified by imaging or biopsy) were screened during the study period. According to the inclusion and exclusion criteria, 8,324 patients were excluded (3,753 patients had HBsAg or anti-HCV positive, 1,225 patients concurrent AIH, PBC, PSC or DILI, 502 patients had incomplete biochemical data, 1858 patients had no data on alcohol consumption, and 986 patients had no liver biopsy). Finally, 713 eligible SLD patients were confirmed by liver biopsy within 7 days of hospitalization and enrolled in this study, including 239 non-drinkers (MASLD), 269 mild-moderate drinkers (MASLD) and 205 excessive drinkers (including 80 MetALD and 125 ALD patients) (Figure 1).

### Patient characteristics

The baseline demographic characteristics, clinical features, and liver histological characteristics of the subjects are reported in Table 1. The median ages were 38, 37, and 42 years in the nondrinking, mild-moderate drinking, and excessive drinking groups, respectively, with a male predominance in each group (51.7%, 88.5%, and 91.7%, *p* < 0.001). Most patients were Han (91.7%) and lived in urban areas (77.0%). A total of 563 (79.0%) patients in the three groups were married (78.2%, 79.2%, and 79.5%, *p* = 0.740). The mild-moderate drinking group had a greater proportion of subjects with fairly better education (83.6%) and part-time or full-time jobs (71.4%) than did the nondrinking and excessive drinking groups. Although fewer than half of the subjects smoked (45.6%), the proportion of current smokers increased with increasing alcohol consumption dose in the three groups (25.1%, 50.6%, and 62.9%, *p* < 0.001). In addition, we observed that the median BMI and the proportion of subjects with obesity were lower in the excessive drinking group than in the non-drinking and mild-moderate drinking groups, while no significant differences were found between the nondrinking and mild-moderate drinking groups with regard to the median BMI value or the proportion of subjects with obesity, hypertension or type 2 diabetes mellitus (T2DM).

**TABLE 1:** Social, demographic, clinical, and liver histology characteristics in patients with biopsy-proven metabolic dysfunction-associated steatotic liver disease.

Characteristic	All subjects (n = 713)	Type of drinkers			*p*-value for trend
		Non-drinkers (n = 239)	Mild-moderate drinkers (n = 269)	Excessive drinkers (n = 205)	
Age (years), median (interquartile range)	39.0 (19.0)	38.0 (22.0)	37.0 (18.0)	42.0 (16.0)	0.079
Gender, n (%)					<0.001
Male	550 (77.1%)	124 (51.7%)	238 (88.5%)	188 (91.7%)	
Ethnicity, n (%)					0.511
Han	654 (91.7%)	220 (92.1%)	249 (92.6%)	185 (90.2%)	
Non-han	59 (8.3%)	19 (7.9%)	20 (7.4%)	20 (9.8%)	
Marital status, n (%)					0.740
Single or divorced	150 (21.0%)	52 (21.8%)	56 (20.8%)	42 (20.5%)	
Married	563 (79.0%)	187 (78.2%)	213 (79.2%)	163 (79.5%)	
Education, n (%)					0.002
<High school	167 (23.4%)	52 (21.8%)	44 (16.4%)	71 (34.6%)	
≥High school	546 (76.6%)	187 (78.2%)	225 (83.6%)	134 (65.4%)	
Employment status, n (%)					0.026
Retired or unemployed	226 (31.7%)	69 (28.9%)	77 (28.6%)	80 (39.0%)	
Part-time or full-time employed	487 (68.3%)	170 (71.1%)	192 (71.4%)	125 (61.0%)	
Neighborhood population density, n (%)					0.119
Urban	549 (77.0%)	197 (82.4%)	195 (72.5%)	157 (76.6%)	
Rural	164 (23.0%)	42 (17.6%)	74 (27.5%)	48 (23.4%)	
Smoking, n (%)					<0.001
Current	325 (45.6%)	60 (25.1%)	136 (50.6%)	129 (62.9%)	
Past or never	388 (54.4%)	179 (74.9%)	133 (49.4%)	76 (37.1%)	
BMI, kg/m^2^	26.0 ± 3.4	26.3 ± 3.2	26.4 ± 3.4	25.3 ± 3.6^a, b^	0.002
Overweight/obesity, n (%)^c^	601 (84.3%)	210 (87.9%)	238 (88.5%)	153 (74.6%)^a^	<0.001
Hypertension, n (%)	125 (17.5%)	35 (14.6%)	45 (16.7%)	45 (22.0%)^a^	0.046
T2DM, n (%)	96 (13.5%)	39 (16.3%)	32 (11.9%)	25 (12.1%)	0.190
Laboratory parameters					
WBC,×10^9^/L	5.0 (2.1)	4.9 (1.5)	4.9 (1.8)	5.5 (3.8)^a^	0.106
HGB, g/L	147.0 (21.0)	146.5 (23.2)	148.0 (20.0)	147.0 (17.8)	0.995
MCV, fL	90.4 ± 5.9	89.7 ± 4.9	89.6 ± 5.6	92.3 ± 6.9^a^	<0.001
PLT, ×10^9^/L	212.0 (79.0)	233.0 (69.0)	212.0 (75.0)^a^	192.5 (72.3)^a^	<0.001
ALT, U/L	76.5 (86.0)	72.0 (80.0)	80.0 (93.0)	75.5 (92.0)^a^	<0.001
AST, U/L	46.0 (43.0)	44.0 (36.0)	41.0 (32.0)	58.5 (97.0)^a^	<0.001
TBIL, μmol/L	13.5 (10.5)	11.7 (6.6)	14.8 (10.7)	16.3 (13.4)^a^	<0.001
ALP, U/L	87.0 (48.0)	87.0 (41.0)	81.0 (37.0)	99.0 (83.3)^a^	<0.001
GGT, U/L	68.0 (108.0)	67.0 (64.0)	65.5 (84.5)	119.0 (265.0)^a^	<0.001
Total cholesterol, mmol/L	4.7 (1.3)	4.8 (1.3)	4.6 (1.2)^a^	4.7 (1.4)	0.619
Triglyceride, mmol/L	2.2 ± 1.6	2.2 ± 1.5	2.2 ± 1.6	2.3 ± 1.7	0.419
HDL-C, mmol/L	1.0 (0.4)	1.1 (0.3)	1.0 (0.3)	1.0 (0.4)	0.856
LDL-C, mmol/L	3.3 (1.0)	3.4 (0.9)	3.0 (1.0)^a^	3.3 (1.1)	0.965
Glucose, mmol/L	5.1 (0.8)	5.1 (1.0)	5.0 (0.9)	5.1 (0.8)	0.952
Histology					
Significant steatosis (≥2), n (%)	412 (57.8%)	184 (77.0%)	174 (64.7%)^a^	54 (26.3%)^a^	<0.001
Significant lobular inflammation (≥2), n (%)	197 (27.6%)	42 (17.6%)	62 (23.1%)	93 (45.4%)^a^	<0.001
Significant ballooning (>1), n (%)	372 (52.2%)	146 (61.1%)	130 (48.3%)^a^	96 (46.8%)^a^	0.002
Significant fibrosis (≥2), n (%)	216 (30.3)	61 (25.5%)	39 (14.5%)^a^	116 (56.6%)^a^	<0.001

^a^ *P* < 0.05 compared to  Non-drinkers.

^b^ *P* < 0.05 compared to Mild-moderate drinkers.

Continuous variables were presented as mean ± SD (standard deviation) if the data conformed to a normal distribution or as median (interquartile range) if the data did not conform to a normal distribution.

^c^ BMI ≥23 kg/m2 in asians.

In terms of laboratory parameters, the mean corpuscular volume (MCV), alanine aminotransferase (ALT), aspartate aminotransferase (AST), alkaline phosphatase (ALP), gamma-glutamyl transpeptidase (GGT) and total bilirubin (TBIL) were significantly different between FLD patients with excessive alcohol use and those without alcohol use but were comparable between mild-moderate drinkers and non-drinkers. In addition, compared to non-drinkers, mild-moderate drinkers presented lower levels of total cholesterol (TC) and low-density lipoprotein cholesterol (LDL-C) but similar levels of triglyceride (TG), high-density lipoprotein cholesterol (HDL-C) and glucose (Table 1).

**FIGURE 2:**
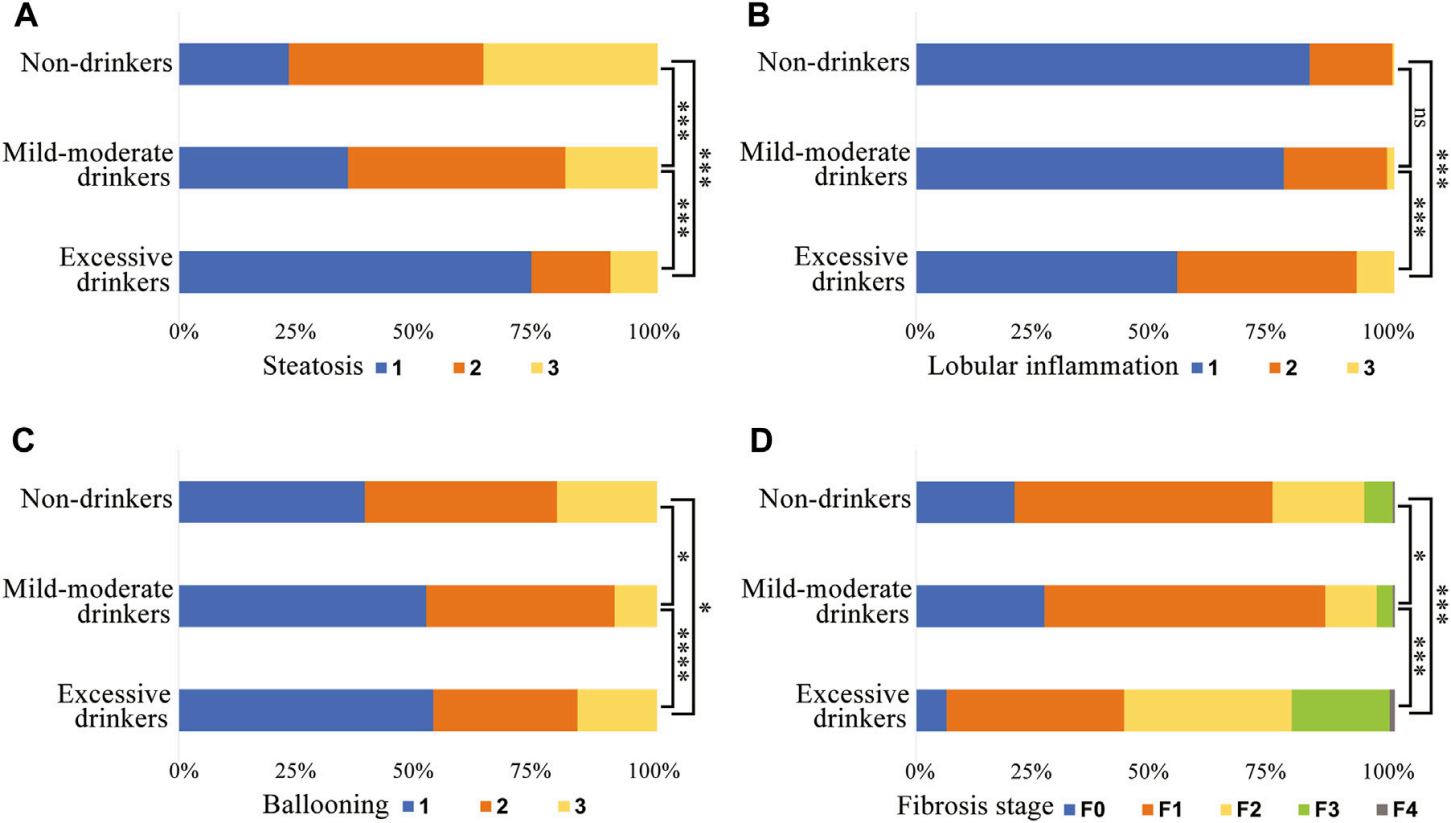
Subject proportions of the severity of **(A)** steatosis, **(B)** lobular inflammation, **(C)** hepatocyte ballooning and **(D)** fibrosis in different alcohol intake groups. *, *p* < 0.05. ***, *p* < 0.001. ****, *p* < 0.0001. ns, not significant.

The proportion of patients who developed severe histological outcomes and the frequency of each histological stage are presented in Table 1; Figure 2. A total of 77.0% of lifetime non-drinkers, 64.7% of mild-moderate drinkers and 26.3% of excessive drinkers developed significant steatosis. The number of patients with mild steatosis gradually increased with increasing alcohol consumption in the three groups (Figure 2A). There was a lower proportion of mild-moderate and excessive drinkers than lifetime nondrinkers for significant ballooning hepatocellular injury (Figure 2C). In contrast, for severe lobular inflammation and fibrosis, there was a significantly greater proportion of excessive drinkers than mild-moderate drinkers and nondrinkers (Figures 2B, D). The mild-moderate drinking group presented an even lower degree of fibrosis than the nondrinking group. (Figure 2D). However, no significant differences in the degree of lobular inflammation were found between mild-moderate drinkers and nondrinkers (Figure 2B).

### The relationship between alcohol consumption and serum lipid and glucose metabolism

According to the stratification of steatosis, the levels of TC and TG were greater in the excessive drinking group, and this association was more obvious in patients in the severe steatosis group than in those in the moderate and/or mild steatosis groups (Supplementary Figures S1A, B). The mild-moderate drinking group presented lower levels of TC and TG, regardless of hepatic steatosis level, in MASLD patients, but the difference was not statistically significant. The level of HDL-C was lower in the mild-moderate drinking group than in the non-drinker group in the mild and/or moderate steatosis group (Supplementary Figure S1C). The mild-moderate drinking group had the lowest LDL-C content among the three drinking groups, and this association was significant in patients in the mild steatosis group (Supplementary Figure S1D). Moreover, the mild-moderate drinking group presented a lower glucose level than the non-drinker group in the moderate steatosis group (Supplementary Figure S1E).

According to the stratification of fibrosis, the level of TC was lower in mild-moderate drinkers than in nondrinkers and excessive drinkers in the moderate steatosis group (Supplementary Figure S2A). There were no significant differences in the TG, LDL-C or glucose levels among the MASLD patients in the three drinking groups, regardless of hepatic steatosis level (Supplementary Figures S2B, D, E). The level of HDL-C was lower in the mild-moderate drinking group than in the non-drinker group in the mild steatosis group (Supplementary Figure S2C).

### Univariate and multivariate logistic regression analysis of risk factors associated with steatosis, lobular inflammation, ballooning and fibrosis

**FIGURE 3:**
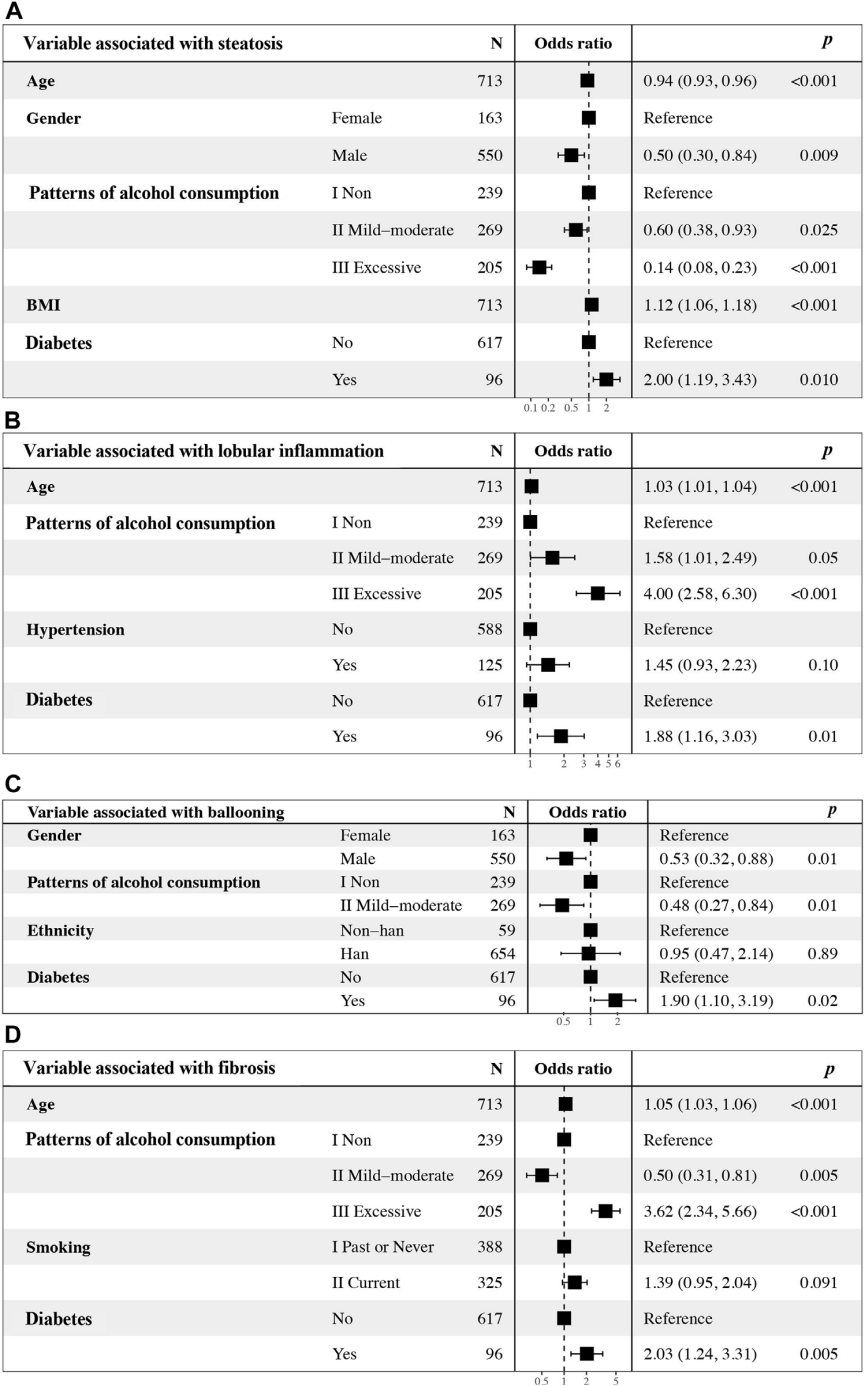
Multivariate logistic regression analyses of alcohol consumption, other clinical factors, and independence associated with **(A)** steatosis, **(B)** lobular inflammation, **(C)** ballooning and **(D)** fibrosis. OR, odds ratio. CI, confidence interval.

Univariate logistic regression analysis indicated that parameters associated with steatosis were age, gender, patterns of alcohol consumption, BMI, and diabetes (Table 2). Subsequent multivariate logistic regression suggested that age (OR = 0.94, 95% CI = 0.93–0.96, *p* < 0.001), gender (OR = 0.50, 95% CI = 0.30–0.84, *p* = 0.009), mild-moderate alcohol consumption (OR = 0.60, 95% CI = 0.38–0.93, *p* = 0.025) and excessive alcohol consumption (OR = 0.14, 95% CI = 0.08–0.23, *p* < 0.001) were all negative factors for advanced hepatic steatosis, while BMI (OR = 1.12, 95% CI = 1.06–1.18, *p* < 0.001) and diabetes (OR = 2.00, 95% CI = 1.19–3.43, *p* = 0.010) may promote steatosis (Figure 3A).

**TABLE 2:** Univariate logistic regression analysis of risk factors associated with hepatic steatosis.

Variables	Or (95% CI)	*p*-value
Age		
≥median (39 years)	0.377 (0.277–0.512)	0.001
< median (39 years)		
Gender		
Male	0.610 (0.420–0.870)	0.008
Female		
Patterns of alcohol consumption		
Excessive drinking	0.110 (0.069–0.160)	0.001
Moderate drinking	0.550 (0.368, 0.808)	0.003
Non drinking		
Ethnicity		
Han	1.261 (0.739–2.150)	0.395
Non-han		
Smoking		
Current	0.854 (0.634–1.151)	0.301
Past or never		
BMI		
≥23 kg/m^2^	1.656 (1.205–2.276)	0.002
<23 kg/m^2^		
Hypertension		
Yes	0.915 (0.620–1.351)	0.657
No		
Diabetes		
Yes	1.631 (1.034–2.575)	0.034
No		

OR, Odds ratio. CI, confidence interval.

Univariate logistic regression analysis indicated that parameters associated with lobular inflammation were age, patterns of alcohol consumption, hypertension, and diabetes (Table 3). Subsequent multivariate logistic regression suggested that all factors identified via univariate analysis, except for hypertension, were associated with advanced lobular inflammation, including age, mild-moderate and excessive alcohol consumption and diabetes. Drinking excessively played a critical role (OR = 4.00, 95% CI = 2.58–6.30, *p* < 0.001) (Figure 3B).

**TABLE 3:** Univariate logistic regression analysis of risk factors associated with lobular inflammation.

Variables	Or (95% CI)	*p*-value
Age		
≥median (39 years)	2.022 (1.441–2.839)	0.001
< median (39 years)		
Gender		
Male	0.760 (0.521–1.122)	0.200
Female		
Patterns of alcohol consumption		
Excessive drinking	3.890 (2.540–6.041)	0.001
Moderate drinking	1.401 (0.910–2.190)	0.100
Non drinking		
Ethnicity		
Han	1.028 (0.565–1.872)	0.927
Non-han		
Smoking		
Current	1.296 (0.933–1.801)	0.122
Past or never		
BMI		
≥23 kg/m^2^	0.960 (0.675–1.363)	0.818
<23 kg/m^2^		
Hypertension		
Yes	1.917 (1.279–2.872)	0.001
No		
Diabetes		
Yes	1.988 (1.273–3.104)	0.002
No		

OR, Odds ratio. CI, confidence interval.

Univariate logistic regression analysis indicated that parameters associated with liver ballooning were gender, mild-moderate alcohol consumption, ethnicity and diabetes (Table 4). Subsequent multivariate analyses revealed that male sex (OR = 0.58, 95% CI = 0.35–0.95, *p* = 0.03) and mild-moderate alcohol consumption (OR = 0.52, 95% CI = 0.29–0.91, *p* = 0.02) were negatively associated with liver ballooning; however, diabetes (OR = 2.02, 95% CI = 1.61–3.43, *p* = 0.01) was a risk factor associated with liver ballooning (Figure 3C).

**TABLE 4:** Univariate logistic regression analysis of risk factors associated with liver ballooning.

Variables	Or (95% CI)	*p*-value
Age		
≥median (39 years)	1.010 (0.990–1.030)	0.200
< median (39 years)		
Gender		
Male	0.460 (0.300–0.710)	0.001
Female		
Patterns of alcohol consumption		
Excessive drinking	0.750 (0.460–1.210)	0.200
Moderate drinking	0.370 (0.220–0.620)	<0.001
Non drinking		
Ethnicity		
Han	1.090 (0.450–2.020)	0.100
Non-han		
Smoking		
Current	0.719 (0.473–1.093)	0.123
Past or never		
BMI		
≥23 kg/m^2^	1.030 (0.970–1.090)	0.400
<23 kg/m^2^		
Hypertension		
Yes	1.080 (0.620–1.800)	0.800
No		
Diabetes		
Yes	1.970 (1.150–3.280)	0.010
No		

OR, Odds ratio. CI, confidence interval.

Univariate logistic regression analysis indicated that parameters associated with hepatic fibrosis were age, patterns of alcohol consumption, smoking, and diabetes (Table 5). Subsequent multivariate analyses revealed that patients with excessive alcohol consumption (OR = 3.62, 95% CI = 2.34–5.66, *p* < 0.001) and diabetes (OR = 2.03, 95% CI = 1.24–3.31, *p* < 0.001) were more likely to have advanced fibrosis; however, mild-moderate alcohol consumption was unexpectedly found to be an independent predictor that was negatively associated with the stage of liver fibrosis (OR = 0.50, 95% CI = 0.31–0.81, *p* = 0.005) compared to lifetime abstinence (Figure 3D).

**TABLE 5:** Univariate logistic regression analysis of risk factors associated with liver fibrosis.

Variables	Or (95% CI)	*p*-value
Age		
≥median (39 years)	2.938 (2.022–3.985)	0.001
< median (39 years)		
Gender		
Male	1.049 (0.720–1.548)	0.789
Female		
Patterns of alcohol consumption		
Excessive drinking	3.802 (2.564–5.711)	0.001
Moderate drinking	0.489 (0.310–0.774)	0.002
Non drinking		
Ethnicity		
Han	1.081 (0.600–1.944)	0.796
Non-han		
Smoking		
Current	1.556 (1.129–2.145)	0.007
Past or never		
BMI		
≥23 kg/m^2^	1.123 (0.795–1.588)	0.510
<23 kg/m^2^		
Hypertension		
Yes	1.372 (0.914–2.059)	0.126
No		
Diabetes		
Yes	2.189 (1.412–3.394)	0.001
No		

OR, Odds ratio. CI, confidence interval.

Furthermore, the non-excessive drinkers, including nondrinkers and mild-moderate drinkers, were divided into two groups according to whether they had T2DM. We assessed the influence of alcohol consumption on fibrosis in non-excessive drinkers with and without T2DM who had biopsy-proven fatty liver disease. In non-excessive drinkers without T2DM, mild-moderate drinking was negatively associated with the stage of liver fibrosis (OR = 0.465, 95% CI = 0.273–0.789, *p* = 0.005) (Supplementary Table S1). However, in non-excessive drinkers with T2DM who had biopsy-proven MASLD, we did not find an association between mild-moderate drinking and liver fibrosis (OR = 0.562, 95% CI = 0.207–1.530, *p* = 0.257) (Supplementary Table S2).

## Discussion

In this study, we first found that the laboratory parameters of liver injury were comparable between mild-moderate drinkers and lifetime non-drinkers, suggesting that mild-moderate alcohol consumption is unlikely to be involved in advanced liver injury. Regarding histological outcomes, no significant differences in the degree of lobular inflammation were found between mild-moderate drinkers and non-drinkers, and the mild-moderate drinking group presented an even lower proportion of patients with severe liver fibrosis than the nondrinking group.

In our cohort, univariate and multivariate logistic regression analyses indicated that mild-moderate drinking was an independent predictor that was negatively associated with the stage of liver fibrosis compared to lifetime abstainers. This finding is consistent with a previous report that modest (1–70 g per week) alcohol consumption, particularly wine consumption in a non-binge pattern, was associated with reduced fibrosis in patients with NAFLD/MASLD (Mitchell et al., 2018). In addition, in a well-characterized population with biopsy-proven NAFLD/MASLD, moderate alcohol consumption was associated with a lower degree of severity, as determined by lower odds of the key features that comprise a diagnosis of steatohepatitis, as well as fibrosis (Dunn et al., 2012). The mechanism by which mild-moderate alcohol consumption might be protective against liver fibrosis in MASLD is uncertain. Yamada et al. (2018) identified a strong suppression of monocytes and their surface molecules in the light alcohol consumer group compared to that in the non-alcohol NAFLD group, indicative that a small amount of alcohol intake may be effective in suppressing macrophage-derived proinflammatory cytokines and, thereby, delaying the progression of steatohepatitis. The endotoxin markers also have been shown to be lower in patients with moderate alcohol consumption (Wong et al., 2015). These studies indicate that mild-moderate alcohol consumption might be protect against liver inflammation and fibrosis in MASLD by exerting anti-inflammatory and antioxidant effects.

The inactive ALDH2 504lys allele occurred mainly in Asian populations (Crabb et al., 2004). It is reported that no association was observed between ADH1B/ALDH2 mutant alleles and hepatic steatosis/fibrosis in patients with NAFLD (Chien et al., 2023). In our previous study (Chang et al., 2018), we found that the prevalence of the common form of the SNP rs671, 504glu (glu/glu) was significantly higher in patients with alcoholic liver disease (ALD) (95.4%) compared to healthy controls (73.7%, *P* < 0.0001). Among controls, 23.7% had heterozygous (glu/lys) genotype compared to 4.6% in those with ALD (*P*< 0.0001). None of the patients with ALD had homozygous lys/lys genotype compared to 2.6% among controls (*p* < 0.05). The allele frequency for 504lys allele in patients with ALD was 2.3% compared to 14.5% in healthy controls (*P* < 0.0001). The major finding in our study is that patients with ALDH2 504lys variant were less associated with ALD compared to those with ALDH2 504glu using both genotypic and allelic analyses. Our findings indicate that ALDH2 504lys may be protected against ALD, in accordance with previous reports (Li et al., 2012; Yokoyama et al., 2013). It is postulated that ALDH2 504lys protects against excessive alcohol use or alcoholism because of unpleasant symptoms secondary to acetaldehyde accumulation. Lazo et al. (2021) found that both a J shaped association between alcohol consumption and hepatic steatosis among those with the CC genotype of PNPLA3, and a higher prevalence of hepatic steatosis among those with PNPLA3 gene G variant. Compared to never drinkers, moderate alcohol drinking was associated with a 48% decreased risk of hepatic steatosis only among those without PNPLA3 G allele (PR = 0.52, 95% CI 0.26–1.05), with no association among those with at least one copy of the PNPLA3 G allele (PR = 1.02, 95% CI 0.68–1.54). These results indicate that keeping alcohol consumption low might offset genetic predisposition to liver disease (Lazo et al., 2021). Rashu et al. (2024) have found that PNPLA3 was with an odds ratio of 6.75 (95% CI 1.29–50.7; *p* = 0.039) risk allele CG/GG versus CC for significant fibrosis in MASLD patients. However, the SNPs in HSD17B13 or TM6SF2 were not individually associated with fibrosis, respectively (Rashu et al., 2024). In contrast, A risk score based on three genetic risk variants (NPLA3:rs738409, SUGP1-TM6SF2:rs10401969, HSD17B13:rs6834314) and diabetes status can provide meaningful risk stratification for cirrhosis in excess drinkers (Whitfield et al., 2022). To overcome this issue, a randomized controlled trial should be performed to evaluate the role of different genetic polymorphism (ALDH2, PNPLA3, TM6SF2) in fibrosis in MASLD patients with mild-moderate alcohol consumption.

In our study, mild-moderate alcohol consumption was associated with less severe fibrosis, steatosis, and ballooning without aggravating lobular inflammation. Several early studies have suggested a liver-protective role of moderate alcohol consumption; however, these studies have been limited by the lack of information on liver histology (Dunn et al., 2008; Gunji et al., 2009; Moriya et al., 2011; Sookoian et al., 2014). Four studies have reported that modest alcohol consumption is associated with lower liver fibrosis, as determined by liver biopsy, in patients with NAFLD/MASLD than in nondrinking patients (Dunn et al., 2012; Hagstrom et al., 2017; Mitchell et al., 2018; Yamada et al., 2018). These studies are consistent with our findings and suggest that variable low levels of alcohol consumption might have a beneficial effect on the improvement of liver fibrosis. A J-shaped association between alcohol consumption and cirrhosis risk was suggested in the Copenhagen study (Becker et al., 1996) and the Nurse’s Health Study (Fuchs et al., 1995). In our study, mild-moderate drinkers had lower serum total cholesterol and LDL-C levels than non-drinkers, which might partly explain the potential benefit of moderate alcohol consumption in individuals with MASLD. Other previous studies are not consistent with our results; some studies have reported that mild or moderate drinking has protective effects on NAFLD or liver histology in NAFLD patients, while other studies have reported no association or even harmful effects. Chang et al. (2019) demonstrated that non-excessive alcohol consumption, especially moderate alcohol consumption, was significantly and independently associated with the worsening of noninvasive markers of fibrosis in NAFLD individuals without liver biopsy. This study is limited by the fact that the stage of liver fibrosis was assessed using noninvasive fibrosis markers and not through tissue biopsy. Li et al. (2012) showed that modest alcohol consumption was associated with less improvement in steatosis (−0.49 vs. −0.30, respectively, *p* = 0.04) and the level of AST (mean change in AST: −7 U/L vs. + 2 U/L, *p* = 0.04) compared to no use of alcohol in a longitudinal analysis of liver biopsies from patients with NAFLD. However, non-drinkers did not have more improvement in fibrosis than modest drinkers (0.06 vs. 0.08, respectively, *p* = 0.85) in that study (Ajmera et al., 2018). Recently, Rice et al. (2022) showed that nonheavy alcohol use was associated with fibrosis and NASH. This study was also limited by the following facts: 1) the degree of liver fibrosis was not assessed by liver biopsy, and 2) the nonheavy alcohol consumption group was not compared with the non-drinking group because non-drinkers were excluded from their study (Rice et al., 2022).

It has been reported that the cumulative incidence of fibrosis progression was significantly greater in participants with T2DM than in participants without T2DM who had biopsy-proven NAFLD (Huang et al., 2023). We also found that T2DM was a significant independent predictor of an increased risk of steatosis, lobular inflammation, ballooning and fibrosis among patients with biopsy-proven MASLD. A previous study reported that T2DM is associated with a significantly greater cumulative incidence of fibrosis progression, possibly related to the stimulating effect of hyperinsulinemia and high glucose levels on hepatic stellate cells (Paradis et al., 2001). Modest alcohol consumption ameliorates metabolic risk factors for MASLD, possibly through a protective mechanism by reducing fasting serum insulin and triglyceride levels (Davies et al., 2002; Howard et al., 2004). However, in non-excessive drinkers with T2DM who had biopsy-proven MASLD, we did not find an association between mild-moderate drinking and liver fibrosis. Additional molecular pathway studies with animal models are warranted to clarify whether and how causal relationships exist.

Additionally, despite being widely recognized as a common cause of fatty liver, the exact impact of alcohol consumption on hepatic steatosis in the MASLD population is elusive (Long et al., 2020; Niezen et al., 2021; Unalp-Arida and Ruhl, 2022). We seemed to observe an association between alcohol consumption and liver steatosis. On the one hand, the median BMI and the proportion of subjects with obesity were lower in the excessive drinking group than in the nondrinking and mild-moderate drinking groups. A gradual decrease in patients with severe steatosis was observed for non-drinkers (77.0%), mild-moderate drinkers (64.7%) and excessive drinkers (26.3%). On the other hand, an inverse association was found between steatosis and both mild-moderate and excessive alcohol consumption in subjects with MASLD according to univariate and multivariate logistic regression analyses. Although the regression analysis revealed that moderate or excessive alcohol consumption was closely related to milder cell steatosis in our collected subjects, we cannot conclude that alcohol use exerted a steatosis-protective effect on patients with MASLD. Although all of the assessed patients had different grades of biopsy-proven steatosis, MASLD patients who presented at the hospital due to alcohol-induced liver damage may have experienced milder steatosis overall than MASLD patients who visited the hospital without alcohol consumption. In other words, because patients with MASLD who drink alcohol usually tend to go to hospitals for examination earlier, the “mild-moderate” and “excessive” drinking groups we divided here presented relatively mild degrees of liver steatosis. In contrast, patients with MASLD who were not drinkers did not consider their condition seriously and visited hospitals for an invasive liver biopsy until steatosis progressed to a severe degree. This led us to observe a greater proportion of patients with severe steatosis in the nondrinking group than in the drinking group and revealed a reverse association between drinking and liver steatosis. However, further studies are required to assess the precise functions of alcohol consumption in MASLD.

As with all previous studies, a limitation of our study is the risk of recall bias due to the nature of alcohol history questionnaires. The quantification of alcohol use by self-reports may be inaccurate. Second, the cross-sectional design cannot address the temporal relationship or causality between mild-moderate alcohol consumption and liver fibrosis, and a prospective cohort study is needed to confirm the reliability of the research results.

In conclusion, our study showed that T2DM was significantly and independently associated with a greater risk of fibrosis among patients with biopsy-proven MASLD, but mild-moderate alcohol consumption was significantly and independently associated with a lower risk of liver fibrosis. In non-excessive drinkers with T2DM who had biopsy-proven MASLD, there was no association between mild-moderate alcohol consumption and liver fibrosis compared to non-drinkers. While our data suggest that a mild-moderate level of alcohol consumption is not associated with severe liver fibrosis in patients with MASLD who have T2DM, alcohol consumption should be avoided in patients with MASLD due to the association with increased risks for advanced liver disease and cancer (Aberg et al., 2020), and further research is needed to establish whether a mild-moderate level of alcohol consumption represents a “healthier” lifestyle effect in patients with MASLD.

## Data Availability

The original contributions presented in the study are included in the article/Supplementary Material, further inquiries can be directed to the corresponding authors.

## Abbreviations

ALT, alanine aminotransferase; AUDIT, Alcohol Use Disorders Inventory Test; AIH, autoimmune hepatitis; AST, aspartate aminotransferase; ALP, alkaline phosphatase; BMI, body mass index; CR, serum creatinine; CI, confidence interval; GGT, gamma-glutamyl transpeptidase; HBV, hepatitis B virus; HCV, hepatitis C virus; HDL-C, high-density lipoprotein cholesterol; LDL-C, low-density lipoprotein cholesterol; MASLD, metabolic dysfunction-associated steatotic liver disease; MCV, mean corpuscular volume; NAFLD, non-alcoholic fatty liver disease; ns, no significance; OR, odds ratio; PLT, platelet; PBC, primary biliary cholangitis; PSC, primary sclerosing cholangitis; TBIL, total bilirubin; TC, total cholesterol; TG, triglyceride; T2DM, type 2 diabetes mellitus; WBC, white blood cell.
