# Supplementary material for: Mild-moderate alcohol consumption and diabetes are associated with liver fibrosis in patients with biopsy-proven MASLD
Source: Front Pharmacol. 2024 Jul 31;15:1437479. doi: 10.3389/fphar.2024.1437479 (PMC11322122; doi:10.3389/fphar.2024.1437479)
Supplement: Supplementary file 2 [file DataSheet1.PDF]

Supplementary figure legends

Supplementary Figure 1. Comparison of lipid and glucose metabolism indexes including (A) TC, (B) TG, (C) HDL-C, (D) LDL-C, and (E) glucose between non-, mild-moderate, excessive drinkers according to the stratification of steatosis. Mild steatosis =steatosis involving 5-33% of hepatocytes. Moderate steatosis=34-66% steatosis. Severe steatosis = >66% steatosis.

Supplementary Figure 2. Comparison of lipid and glucose metabolism indexes including (A) TC, (B) TG, (C) HDL-C, (D) LDL-C, and (E) glucose between non-, mild-moderate, excessive drinkers according to the stratification of fibrosis. Mild fibrosis = fibrosis stage 0-1. Moderate fibrosis = fibrosis stage 2. Severe fibrosis = fibrosis stage 3-4.

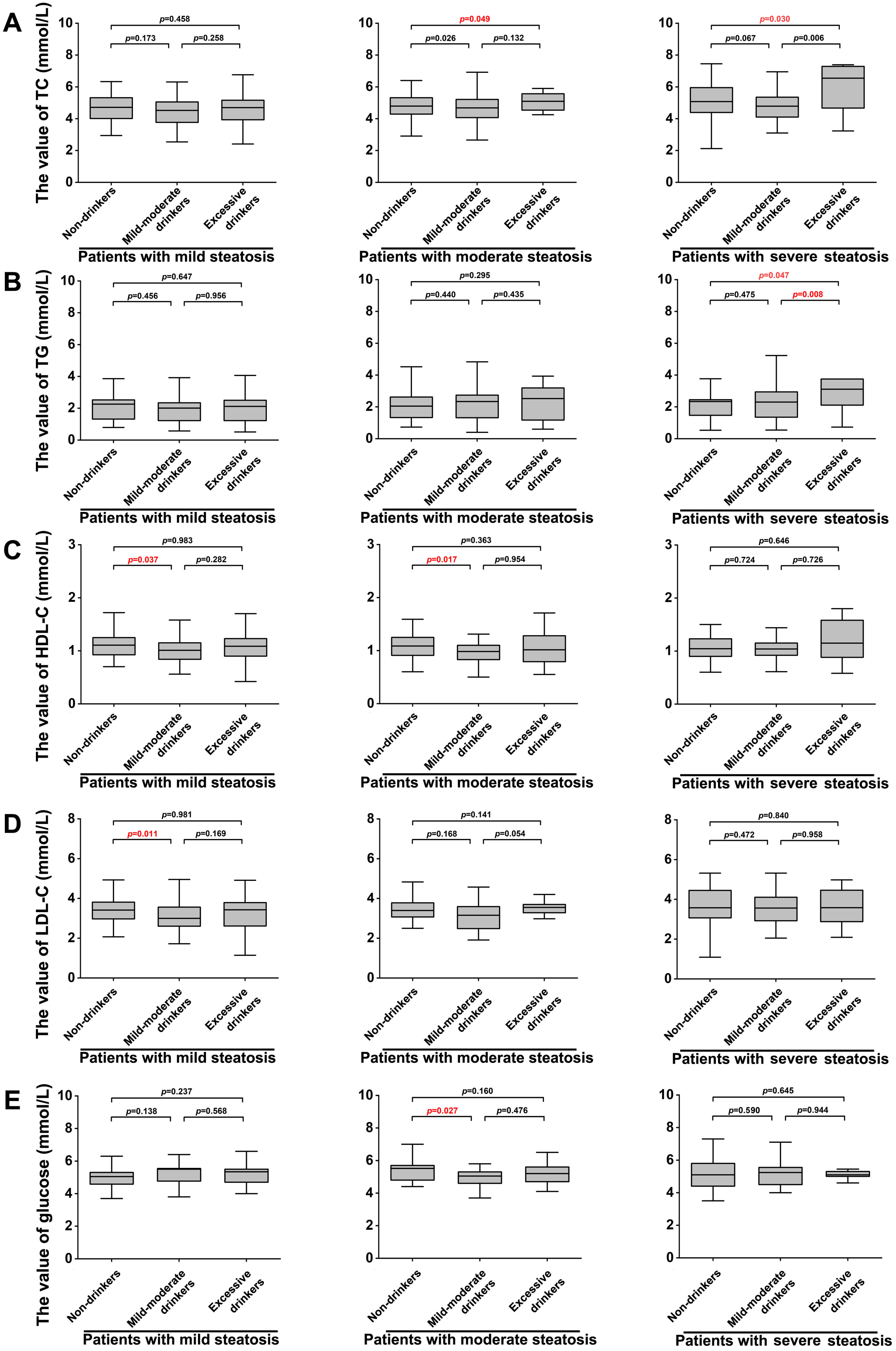
A
B
C
D
E
The value of TC (mmol/L)
The value of TG (mmol/L)
The value of HDL-C (mmol/L)
The value of LDL-C (mmol/L)
The value of glucose (mmol/L)
Non-drinkers
Mild-moderate drinkers
Excessive drinkers
Patients with mild steatosis
Patients with moderate steatosis
Patients with severe steatosis
p=0.458
p=0.173
p=0.258
p=0.049
p=0.026
p=0.132
p=0.030
p=0.067
p=0.006
p=0.647
p=0.456
p=0.956
p=0.295
p=0.440
p=0.435
p=0.047
p=0.475
p=0.008
p=0.983
p=0.037
p=0.282
p=0.363
p=0.017
p=0.954
p=0.646
p=0.724
p=0.726
p=0.981
p=0.011
p=0.169
p=0.141
p=0.168
p=0.054
p=0.840
p=0.472
p=0.958
p=0.237
p=0.138
p=0.568
p=0.160
p=0.027
p=0.476
p=0.645
p=0.590
p=0.944

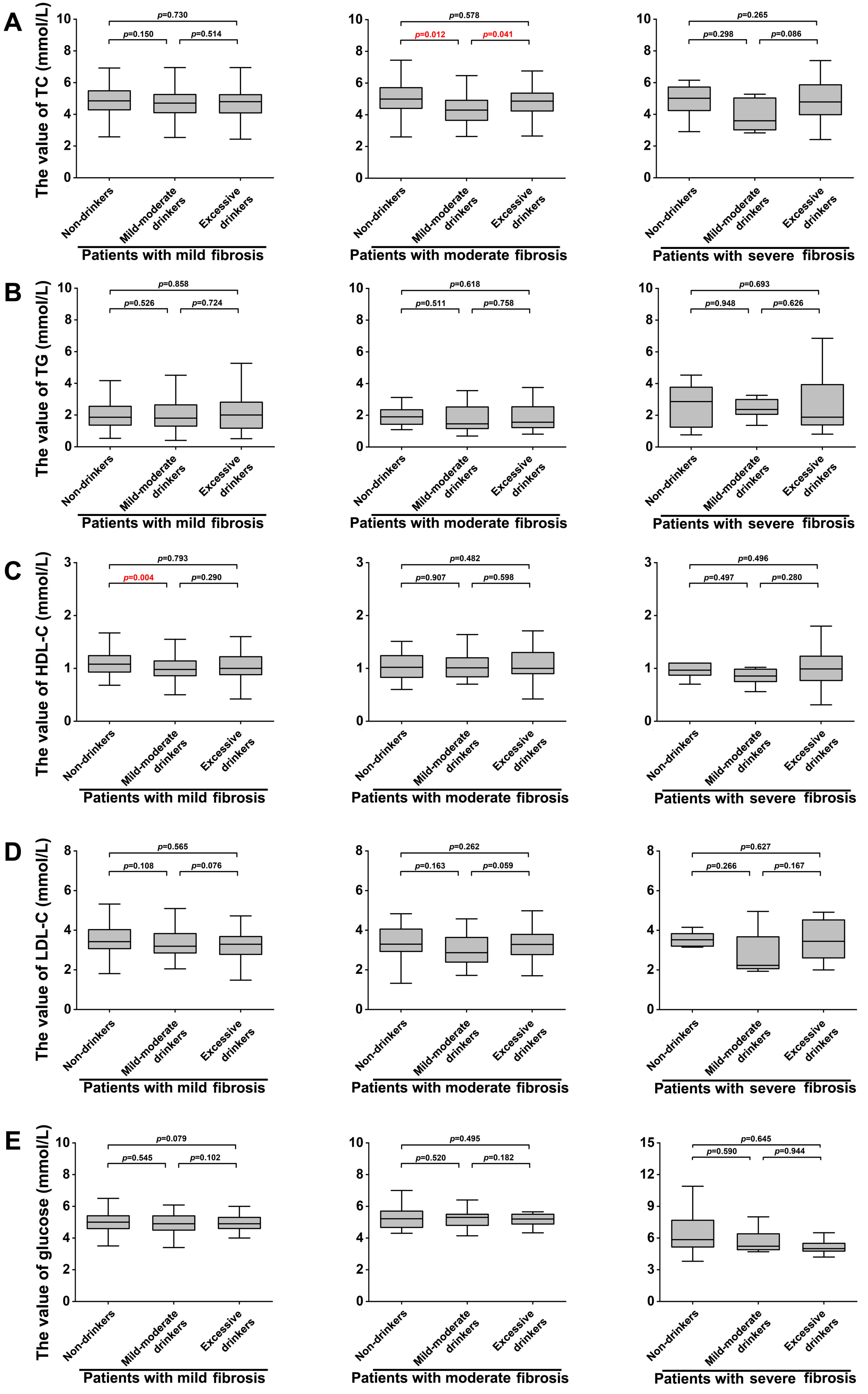
A
The value of TC (mmol/L)
p=0.730
p=0.150
p=0.514
p=0.578
p=0.012
p=0.041
p=0.265
p=0.298
p=0.086
B
The value of TG (mmol/L)
p=0.858
p=0.526
p=0.724
p=0.618
p=0.511
p=0.758
p=0.693
p=0.948
p=0.626
C
The value of HDL-C (mmol/L)
p=0.793
p=0.004
p=0.290
p=0.482
p=0.907
p=0.598
p=0.496
p=0.497
p=0.280
D
The value of LDL-C (mmol/L)
p=0.565
p=0.108
p=0.076
p=0.262
p=0.163
p=0.059
p=0.627
p=0.266
p=0.167
E
The value of glucose (mmol/L)
p=0.079
p=0.545
p=0.102
p=0.495
p=0.520
p=0.182
p=0.645
p=0.590
p=0.944
Non-drinkers
Mild-moderate drinkers
Excessive drinkers
Patients with mild fibrosis
Patients with moderate fibrosis
Patients with severe fibrosis

Supplementary Table 1. Factors for fibrosis in non-excessive drinkers without T2DM：Univariate and Multivariate analysis.

	Significant fibrosis (Stage 2-4) (n=75)	Mild fibrosis (Stage 0-1) (n=362)	Odds ratio (OR, 95 % CI)	*p* value
**Univariate analysis**				
**Age**				
≥median (39 years)	51(26.7%)	140(73.3%)	3.370 (1.985-5.721)	<0.001
< median (39 years)	24(9.8%)	222(90.2%)		
**Gender**				
Female	29(24.2%)	91(75.8%)	1.877 (1.114-3.164)	0.017
Male	46(14.5%)	271(85.5%)		
**Alcohol consumption**				
Mild-moderate Drinkers	30(12.7%)	207(87.3%)	0.499 (0.301-0.829)	0.007
Non-drinkers	45(22.5%)	155(77.5%)		
**Ethnicity**				
Han	65(16.1%)	339(83.9%)	0.441 (0.200-0.970)	0.037
Non-han	10(30.3%)	23(69.7%)		

**Marital status**				
Single or divorced	13(12.7%)	89(87.3%)	0.643 (0.338-1.225)	0.177
Married	62(18.5%)	273(81.5%)		
**Education**				
<High School	25(21.6%)	91(78.4%)	1.489 (0.872-2.544)	0.144
≥High School	50(15.6%)	271(84.4%)		
**Employment status**				
Retired or unemployed	29(20.6%)	112(79.4%)	1.407 (0.840-2.356)	0.193
Part-time or full-time employed	46(15.5%)	250(84.5%)		
**Neighborhood Population Density**				
Urban	60(17.7%)	279(82.3%)	1.119 (0.642-2.205)	0.580
Rural	15(15.3%)	83(84.7%)		
**Smoking**				
Past or never	22(13.2%)	145(86.8%)	0.621 (0.362-1.066)	0.082
Current	53(19.6%)	217(80.4%)		

**BMI**				
≥23 kg/m^2	71(18.7%)	309(81.3%)	3.044 (1.067-8.687)	0.037
<23 kg/m^2	4(7.0%)	53(93.0%)		
**Hypertension**				
Yes	11(17.7%)	51(82.3%)	1.048 (0.518-2.121)	0.896
No	64(17.1%)	311(82.9%)		
**Multivariate analysis**				
**Age**				
≥median (39 years)	51(26.7%)	140(73.3%)	3.541 (2.057-6.097)	<0.001
< median (39 years)	24(9.8%)	222(90.2%)		
**Alcohol consumption**				
Mild-moderate Drinkers	30(12.7%)	207(87.3%)	0.468 (0.276-0.795)	0.005
Non-drinkers	45(22.5%)	155(77.5%)		
**BMI**				
≥23 kg/m^2	71(18.7%)	309(81.3%)	3.214 (1.096-9.425)	0.033

<23 kg/m^2	4(7.0%)	53(93.0%)	

Supplementary Table 2. Factors for fibrosis in non-excessive drinkers with T2DM：Univariate and Multivariate analysis.

	Significant fibrosis (Stage 2-4) (n=25)	Mild fibrosis (Stage 0-1) (n=46)	Odds ratio (OR, 95 % CI)	*p* value
**Univariate analysis**				
**Age**				
≥median (39 years)	20(40.8)	29(59.2)	2.345 (0.744-7.393)	0.140
< median (39 years)	5(22.7)	17(77.3)		
**Gender**				
Female	8(30.8%)	18(69.2%)	0.732 (0.262-2.046)	0.551
Male	17(37.8%)	28(62.2%)		
**Alcohol consumption**				
Mild-moderate Drinkers	9(28.1%)	23(71.9%)	0.562 (0.207-1.530)	0.257
Non-drinkers	16(41.0%)	23(59.0%)		
**Ethnicity**				
Han	24(36.9%)	41(63.1%)	2.927 (0.323-26.556)	0.414
Non-han	1(16.7%)	5(83.3%)		

**Marital status**				
Single or divorced	3(42.9%)	4(57.1%)	1.432 (0.294-6.975)	0.691
Married	22(34.4%)	42(65.6%)		
**Education**				
<High School	9(13.6%)	15(86.4%)	1.162 (0.418-3.234)	0.773
≥High School	16(9.7%)	31(90.3%)		
**Employment status**				
Retired or unemployed	10(47.6%)	11(52.4%)	2.121 (0.743-6.052)	0.156
Part-time or full-time employed	15(30.0%)	35(70.0%)		
**Neighborhood Population Density**				
Urban	20(12.0%)	33(88.0%)	1.576 (0.488-5.084)	0.445
Rural	5(4.6%)	13(95.4%)		
**Smoking**				
Past or never	9(28.1%)	23(71.9%)	0.562 (0.207-1.530)	0.257
Current	16(41.0%)	23(59.0%)		

**BMI**				
≥23 kg/m^2	25(36.8%)	43(63.2%)	1.650 (0.465-5.851)	0.547
<23 kg/m^2	0(0%)	3(100%)		
**Hypertension**				
Yes	8(44.4%)	10(55.6%)	1.694 (0.567-5.059)	0.342
No	17(32.1%)	36(67.9%)		
**Multivariate analysis**				
univariate analyses showed that no factor had an independent impact on fibrosis in non-excessive drinkers with T2DM				
